# The 4-D landscape of the inflammatory response

**DOI:** 10.1186/1756-8935-6-S1-P64

**Published:** 2013-03-18

**Authors:** Argyris Papantonis

**Affiliations:** 1 Sir William Dunn School of Pathology, University of Oxford, Oxford, OX13RE, UK; 2 Center for Molecular Medicine, University of Cologne, Cologne, D-50931, Germany

## Background

It is widely accepted that chromatin ‘responds’ to physiological cues via protein:DNA interactions and nucleosome rearrangement [1,2], and that transcription plays a key role in its higher-order organization [3]. What remains elusive is how the nuclear landscape reshapes, in 3-D space and time, to facilitate such responses to unfold.

## Materials and methods

We add tumour necrosis factor α (TNFα) to primary human endothelial cells and induce the inflammatory cascade; this is orchestrated by the transcription factor NF-κB [4]. We monitor the response for 0-85 min post-induction using ChIP nucleosome-positioning studies, and chromosome conformation capture, all coupled to next-generation sequencing. We also apply a new approach, where the isolation of ‘transcription factories’ [5] is followed by RNA-seq to uncover nascent transcriptomes.

## Results

First, we redefine early, intermediate, late, and oscillating TNFα-responsive genes, based on changing levels of nascent RNA. We then examine how these co-associate in specialized ‘factories’, some of which further specialize in transcribing responsive non-coding genes [6]. Contacts are driven by NF-κB, and evolve as genes are differentially turned on and off over time. We also monitor nucleosome rearrangements genome-wide; these correlate with poised promoters before induction, and with nucleosome depletion as a result of transcriptional activation, NF-κB binding, enhancer activity in TNFα-stimulated chromosomal domains.

## Conclusions

We provide evidence for a prompt, within <30 min, reshaping of the genome in response to inflammation. This entails *de novo* associations of co-regulated coding and non-coding sequences in specialized 3-D networks that evolve over time, as well as extensive nucleosome depletion. We expect all extracellular cues to signal through analogous specialized networks and reassess our parsimonious model [7] for transcriptional regulation accordingly.
